# Circulating Aryl Hydrocarbon Receptor Is Associated with Latent Tuberculosis Infection in Patients with Type 2 Diabetes

**DOI:** 10.3390/ijms26115384

**Published:** 2025-06-04

**Authors:** Yu-Cheng Cheng, Wei-Chang Huang, Yu-Hsuan Li, Shin-Shin Liu, Meei-Ling Sheu, I-Te Lee

**Affiliations:** 1 Division of Endocrinology and Metabolism, Department of Internal Medicine, Taichung Veterans General Hospital, Taichung 407219, Taiwan; fererro1552@vghtc.gov.tw (Y.-C.C.); brightlight@vghtc.gov.tw (Y.-H.L.); 2 Institute of Biomedical Sciences, National Chung Hsing University, Taichung 402002, Taiwan; 3 School of Medicine, National Yang Ming Chiao Tung University, Taipei 112304, Taiwan; 4 Division of Pulmonary Immunology & Infectious Disease, Department of Chest Medicine, Taichung Veterans General Hospital, Taichung 407219, Taiwan; huangweichangtw@dragon.nchu.edu.tw (W.-C.H.); athena@vghtc.gov.tw (S.-S.L.); 5 School of Medicine, Chung Shan Medical University, Taichung 402306, Taiwan; 6 Department of Post-Baccalaureate Medicine, College of Medicine, National Chung Hsing University, Taichung 402002, Taiwan; 7 Department of Computer Science & Information Engineering, National Taiwan University, Taipei 106319, Taiwan; 8 Nursing Department, Taichung Veterans General Hospital, Taichung 407219, Taiwan

**Keywords:** aryl hydrocarbon receptor, latent tuberculosis, type 2 diabetes

## Abstract

Latent tuberculosis infection (LTBI) is prevalent in patients with type 2 diabetes. We aimed to examine the relationship between serum levels of aryl hydrocarbon receptor (AhR) and LTBI in patients with type 2 diabetes. In this cross-sectional study, patients with type 2 diabetes were screened for LTBI using the QuantiFERON-TB (QFT) test. Of 543 patients screened for LTBI, 133 (24.5%) were QFT-positive. The QFT-positive patients had higher AhR levels than the QFT-negative patients (44.6 [interquartile range: 25.4–58.6] pg/mL vs. 37.8 [interquartile range: 17.4–55.0] pg/mL; *p* = 0.004). According to the receiver operating characteristic curve, the area under the curve was 0.584 (95% confidence interval: 0.528–0.639; *p* = 0.004) and the optimal cutoff value for serum AhR levels was of 37.7 pg/mL for differentiating a QFT-positive result. By a multivariable logistic regression analysis, the patients with high AhR levels had a greater risk of being QFT positive than those with low AhR levels (odds ratio = 1.902, 95% confidence interval: 1.254–2.886; *p* = 0.003). In conclusion, in patients with type 2 diabetes, a high serum AhR level was associated with LTBI.

## 1. Introduction

Tuberculosis (TB) caused by uncontrolled Mycobacterium tuberculosis (Mtb) infection is associated with an increased risk of long-term mortality and remains a heavy burden on global health [1]. Latent tuberculosis infection (LTBI), a TB immunoreactive response without clinically manifested active TB, is associated with a potential risk of developing active TB [2]. LTBI screening for preventive treatment is recommended to reduce the risk of active TB development [3].

LTBI is associated with diabetes mellitus [4]. Individuals with diabetes mellitus have a significantly greater prevalence of LTBI compared with those without [5]. In Taiwan, the prevalence of LTBI, based on the QuantiFERON-TB Gold In-Tube (QFT-GIT) method, is 25.8% in outpatients with diabetes mellitus and aged over 50 years [6]. Diabetes mellitus is also a potential factor for TB reactivation in patients with LTBI [7]. In clinical observations, LTBI has been reported to increase the risk of cardiovascular disease (CVD) [8], but this risk can be reduced by LTBI treatment [9]. The chronic inflammation induced by monocyte alterations may be involved in the underlying pathways between LTBI and CVD [10], but the actual mechanism is still not well understood [11].

Aryl hydrocarbon receptor (AhR) is a ligand-dependent transcription factor of the basic helix–loop–helix family that regulates downstream responses to environmental stimulation [12]. Dioxin (2,3,7,8-tetrachlorodibenzop-dioxin, TCDD), an environmental pollutant, is one of the most notorious AhR ligands [13]. After ligand binding, the AhR/chaperone/ligand complex can translocate from the cytoplasm into the nucleus and dissociate from chaperones to form a DNA-bound complex with the AhR nuclear translocator (ARNT) protein [14]. The AhR–ARNT complex further affects the promoters of target genes, including the cytochrome P450 (CYP) enzymes CYP1A1 and CYP1B1 [14]. An in vitro study of cultured monocytic cells revealed that the activation of AhR can induce the release of several inflammatory mediators, including interleukin (IL)-1β and tumor necrosis factor (TNF)-α [15]. AhR activation also promotes oxidative stress to induce the expression of monocyte chemoattractant protein-1 in cultured human umbilical vein endothelial cells [16].

It has been reported that Mtb infection can induce AhR-driven immune responses [17]. Moreover, a high-fat diet can induce vascular AhR protein expression in mice [18], and serum AhR levels are higher in individuals with overweight or obesity than in those of normal weight [19]. Circulating AhR levels, considered a biomarker of CVD, were recently reported to be correlated with the thickness of epicardial adipose tissue [20]. Because AhR activation and LTBI are potential risk factors for CVD development, we hypothesized that circulating AhR levels would be increased in patients with LTBI. In this study, therefore, we aimed to detect LTBI among outpatients with type 2 diabetes and to assess the serum AhR levels between patients with and without LTBI.

## 2. Results

**Figure 1:**
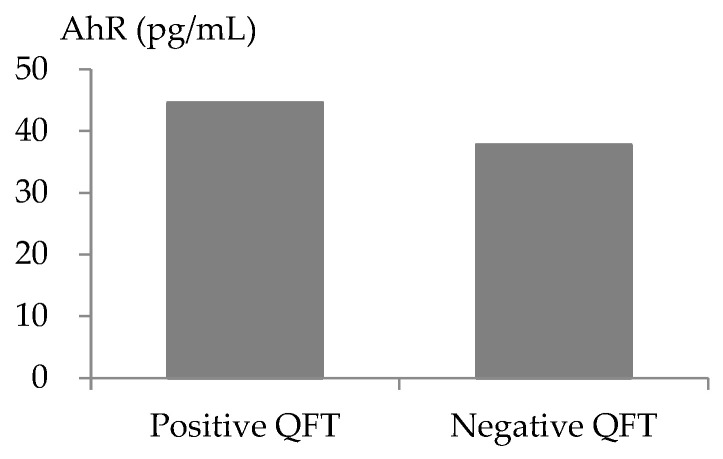
A significant difference in the serum levels of aryl hydrocarbon receptor (AhR) between patients with a positive QuantiFERON-TB (QFT) test result and with a negative one (44.6 [interquartile range: 25.4, 58.6] pg/mL vs. 37.8 [interquartile range: 17.4, 55.0] pg/mL; *p* = 0.004).

Among the 543 patients included in the present study, 133 (24.5%) had a QFT-positive result, and 410 (75.5%) had a QFT-negative result. The AhR levels were 44.6 (interquartile range [IQR: 25.4, 58.6]) pg/mL in the QFT-positive patients and significantly higher than that (37.8 [IQR: 17.4, 55.0] pg/mL) in the QFT-negative patients (*p* = 0.004, Figure 1). The characteristics of the patients with QFT-positive or QFT-negative results are shown in Table 1. Compared with the QFT-negative patients, the QFT-positive patients were older (66.0 [IQR: 60.0, 72.0] years vs. 61.0 [IQR: 55.0, 68.0] years, *p* < 0.001) and had a longer diabetes duration (13.0 [IQR: 8.0, 20.0] years vs. 12.0 [IQR: 7.0, 17.0] years, *p* = 0.024), lower low-density lipoprotein (LDL) cholesterol levels (2.1 [IQR: 1.7, 2.6] mmol/L vs. 2.2 [IQR: 1.8, 2.8] mmol/L, *p* = 0.049), a lower estimated glomerular filtration rate (eGFR) (67.0 [IQR: 53.6, 81.5] mL/min per 1.73 m^2^ vs. 71.9 [IQR: 59.8, 85.8] mL/min per 1.73 m^2^, *p* = 0.001), a lower proportion of sulfonylurea use (32.3% vs. 43.4%, *p* = 0.031), and a lower proportion of sodium–glucose cotransporter 2 (SGLT2) inhibitor use (9.0% vs. 16.6%, *p* = 0.046). There was no significant difference in BMI; systolic blood pressure (BP), diastolic BP, fasting glucose, glycated hemoglobin (HbA1c), total cholesterol, high-density lipoprotein (HDL) cholesterol, triglyceride, alanine aminotransferase (ALT), or C-reactive protein (CRP) levels; sex; hypertension; current smoking status; coronary artery disease (CAD) history; increased urine albumin–creatinine ratio (UACR); or the use of antiplatelet agents, statins, antihypertensive medications, insulin, glucagon-like peptide-1 receptor agonists (GLP-1 RAs), glinides, metformin, dipeptidyl peptidase-4 (DPP4) inhibitors, thiazolidinediones, and α-glucosidase inhibitors between patients in the QFT-positive and QFT-negative groups.

To assess the associations between AhR and the assessed clinical characteristics, the serum AhR levels were compared between the dichotomous groups of clinical characteristics (Table 2). Patients aged ≥63 years had higher AhR levels than those aged <63 years (42.6 [IQR: 21.5, 58.1) pg/mL vs. 37.5 [IQR: 17.4, 54.3] pg/mL, *p* = 0.013). Patients with chronic kidney disease (CKD) had higher AhR levels than those without CKD (42.8 [IQR: 22.9, 59.2] pg/mL vs. 39.4 [IQR: 18.0, 55.1] pg/mL, *p* = 0.043). Patients with a UACR ≥ 30 mg/g had higher AhR levels than those with a UACR < 30 mg/g (42.1 [IQR: 21.9, 58.7] pg/mL vs. 39.7 [IQR: 16.8, 54.3) pg/mL, *p* = 0.032). Patients who used statins had lower AhR levels (38.4 [IQR: 17.4, 55.0] pg/mL vs. 44.3 [23.6, 58.1] pg/mL, *p* = 0.013) than those who did not use statins. Patients treated with DPP4 inhibitors had higher AhR levels than those not treated with DPP4 inhibitors (42.3 [IQR: 23.6, 58.3] pg/mL vs. 37.5 [IQR: 17.4, 54.3] pg/mL, *p* = 0.017).

**Figure 2:**
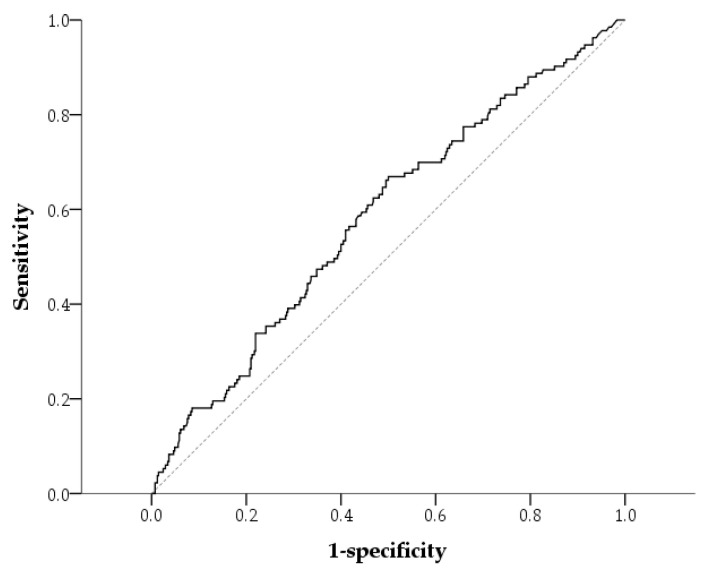
Receiver operating characteristic (ROC) curve (solid line) for differentiating a positive QuantiFERON-TB result from serum levels of aryl hydrocarbon receptor. The area under the curve was 0.584 (95% CI: 0.528–0.639; *p* = 0.004). The dotted diagonal line represents the line of no discrimination.

According to the receiver operating characteristic (ROC) curve (Figure 2), a high serum AhR level was significantly associated with a QFT-positive result (area under the curve = 0.584, 95% confidence interval [CI]: 0.528–0.639; *p* = 0.004). The optimal cutoff value for serum AhR levels was 37.7 pg/mL, which provided a sensitivity of 66.9%, specificity of 50.0%, positive predictive value of 30.3%, and negative predictive value of 82.3% for differentiating a QFT-positive result. We divided the patients into the high-AhR and low-AhR groups using a cutoff value of 37.7 pg/mL. We also included age and CKD in the multivariable logistic regression analyses because they were significantly associated with both a QFT-positive result (based on Table 1) and AhR levels (based on Table 2). Patients in the high-AhR group had a significantly greater risk of a QFT-positive result than those in the low-AhR group according to the multivariable logistic regression analysis (odds ratio [OR] = 1.902, 95% CI: 1.254–2.886, *p* = 0.003) after adjusting for age and CKD. In addition to serum AhR, age was an independent risk factor for a QFT-positive result (OR = 2.258, 95% CI: 1.454–3.507 for patients aged ≥63 years old compared with those < 63 years old; *p* < 0.001; Table 3).

Since age is an independent risk factor for a positive QFT result, we stratified the enrolled patients by the median (63 years) of age. The serum AhR levels ≥ 37.7 pg/mL remained to be an independent risk factor both in patients aged <63 years (OR = 2.218, 95% CI: 1.126–4.371; *p* = 0.021) and in those aged ≥63 years (OR = 1.717, 95% CI: 1.010–2.918; *p* = 0.046; Table 4) after adjusting for CKD.

## 3. Discussion

Our main finding in the present study was that high serum AhR levels were significantly associated with LTBI in patients with type 2 diabetes. AhR signaling plays an important role in the immune responses of barrier organs, including the lungs, against infection [21]. AhR protein expression and AhR activation are both enhanced in cultured THP-1 cells by Mtb infection [17]. The naphthoquinone phthiocol, a pigmented virulence factor from Mtb, has been reported to bind to AhR and induce the transcription of AhR-activating genes [22]. Our findings provide evidence that LTBI is associated with an increase in circulating AhR levels in patients with type 2 diabetes. Therefore, LTBI may have a potential effect on the activation of the AhR signaling pathway.

Numerous studies have indicated that AhR may play a crucial role in the pathogenesis of CVD [23]. Exposure to exogenous ligands of AhR, such as dioxin (2,3,7,8-tetrachlorodibenzop-dioxin, TCDD), increases the risk of ischemic heart disease and cardiovascular mortality [24]. Inflammation activated by AhR signaling pathways can promote the development of atherosclerotic CVD [23]. Wu et al. [25] reported that AhR activation by TCDD promoted vascular inflammation and atherosclerosis in an animal study. Coplanar polychlorinated biphenyls, other environmental contaminants that act as agonists of AhR, can induce oxidative stress and vascular cell adhesion molecule 1 expression to disrupt endothelial barrier function via the AhR/NF-κB signaling pathway in cultured endothelial cells from pulmonary arteries [26]. However, we only present an increased serum AhR level, but not an activated AhR pathway in the present study.

It has been reported that plasma AhR levels are associated with the thickness of epicardial adipose tissue [20]. In addition to a significant association between the AhR polymorphism and CAD, the AhR mRNA levels from mononuclear cells of peripheral blood are significantly higher in patients with CAD than in those without CAD [27]. Therefore, investigating the role of AhR overexpression in cardiovascular risk is important. LTBI increases cardiovascular risk, and completing the treatment of LTBI can significantly reduce the occurrence of cardiovascular events [9]. However, the underlying mechanism between circulating AhR and cardiovascular risk remains unclear. In patients with atopic dermatitis, the protein levels in serum, mRNA in peripheral blood mononuclear cells, and tissue expression in skin lesions of AhR were all increased [28]. Hu et al. reported that serum AhR levels and AhR expression in the kidneys are both increased by tryptophan-induced CKD, which is associated with the activation of the AhR pathway and reflected by the upregulation of CYP1A1 and CYP1B1 in the kidney tissue of mice [29]. Therefore, circulating AhR levels can be a clinical biomarker associated with the activation of the AhR pathway, but the exact mechanism needs further investigations. The treatment of TB may attenuate the inflammatory response and decrease the levels of circulating inflammatory cytokines [30]. Since the overexpression of the AhR/NF-κB signaling pathway is associated with oxidative stress and endothelial dysfunction [26], further studies investigating the treatment target of AhR are warranted in patients with LTBI.

The AhR pathway may be associated with glucose homeostasis [31]. It has been reported that serum AhR levels are inversely correlated with β-cell function in patients without known diabetes mellitus [32]. In the present study, however, serum AhR levels were not significantly associated with fasting glucose or HbA1c levels in patients with known diabetes. The response of the immune-endocrine network to latent TB in individuals with known diabetes is obviously different from that in individuals without known diabetes [33]. Aravindhan et al. [34] reported that the prevalence of LTBI was significantly higher in patients with known diabetes than that in patients with prediabetes or newly diagnosed diabetes. Insulin resistance (IR) may induce several cytokines to activate macrophages and fight against Mtb infection, but this beneficial effect of IR-LTBI antagonism is lost in individuals in patients with a chronic status of diabetes [33]. Therefore, the conditions of diabetes–TB synergy with hyperglycemia and poor TB immunity are observed in patients with known diabetes [33,34].

In a cross-sectional study in patients with diabetes mellitus at a regional hospital, Chang et al. [6] reported that age was an independent risk factor for LTBI in patients with diabetes; however, types of glucose-lowering medications were not significantly associated with LTBI. Although metformin, but not sulfonylurea, was reported to prevent active TB in a meta-analysis of clinical studies [35], Yu et al. [36] reported that metformin could reduce the risk of active TB but not the risk of LTBI. Similarly, metformin was not significantly associated with the QFT-positive result in the present study. Interestingly, we found that the use of sulfonylurea was associated with a low risk of the QFT-positive result in the present study. Sulfonylurea can inhibit acetohydroxyacid synthase (AHAS) from Mycobacterium avium [37]. AHAS catalyzes the first step in the biosynthesis of branched-chain amino acids needed for survival in microorganisms, and this pathway can be a potential target for anti-TB therapy [34]. Sulfonylureas exhibit significant potential against drug-resistant Mtb on the basis of in vitro and intracellular assessments [38]. However, the use of sulfonylurea was not significantly associated with serum AhR levels in this study.

Although an overexpression of AhR is associated with cardiovascular risk, Turco et al. [39] reported that statins could activate the AhR pathway and decrease the inflammatory response in lipopolysaccharide-treated macrophage cells. In the present study, the serum AhR levels were significantly lower in patients who used statins than those who did not use. However, we did not assess the activation of the AhR pathway in this study and did not investigate the association between serum AhR levels and subsequent atherosclerosis. In Mtb-infected macrophages, an activated AhR pathway can induce the secretion of IL-1β, an important inflammatory cytokine associated with atherosclerosis [17,40]. However, Hu et al. reported that serum IL-1β levels might not be significantly correlated with the AhR expression in peripheral blood mononuclear cells [28]. Similarly, serum AhR levels were not significantly associated with CRP levels in the present study.

There are several limitations to the present study. First, we did not directly investigate the mechanisms underlying the relationship between AhR and LTBI. Second, we did not assess the pathophysiological increase in the secretion of the AhR protein from cells into the peripheral blood in patients with LTBI. Hu et al. [28] reported that the serum levels of AhR and CYP1A1 are increased in patients with atopic dermatitis and that disease severity is significantly correlated with AhR expression in peripheral blood mononuclear cells. However, we cannot speculate whether the circulating AhR level is considered the same as the activated AhR signaling pathway. Third, we could not clarify the causal relationship between LTBI and serum AhR levels in the cross-sectional study. Fourth, we cannot exclude other confounding factors which we did not assessed in the present study, as several exogenous factors could activate the AhR signaling pathway [41,42]. Finally, only patients with type 2 diabetes with poor glucose control were enrolled in the study, so our findings may not be generalizable to other populations.

## 4. Materials and Methods

### 4.1. Study Design and Population

In this cross-sectional study, patients aged ≥45 years were included for assessments if they had been diagnosed with diabetes mellitus and had at least one HbA1c level > 9.0% within the previous year according to their medical records. Patients were initially excluded if they had a medical history of active TB, a history of being immunocompromise or using immunosuppressors, or were currently pregnant. After baseline anthropometric characteristics were measured, blood samples and urine samples were collected in the morning after an overnight fast from patients who fulfilled the selection criteria. The blood samples were prepared for assessments of HbA1c, plasma glucose, and serum creatinine levels; lipid profiles; and ALT, CRP, and AhR levels. Moreover, patients were excluded from the present study if the following conditions applied: a diabetic diagnosis other than type 2 diabetes, an acute infection with CRP levels > 60 mg/L, or end-stage renal disease with an eGFR < 15 mL/min per 1.73 m^2^.

### 4.2. Measurements

After enrollment, baseline anthropometric characteristics were measured, and blood samples and urine samples were collected in the morning after an overnight fast. Medical and personal histories were recorded. The current use of glucose-lowering medications, antihypertensive medications, antiplatelet agents, and statins was recorded.

LTBI was assessed using the QFT-GIT (Qiagen, Valencia, CA, USA). The concentrations of serum lipids, creatinine, ALT, and CRP were measured using commercial kits (Beckman Coulter, Fullerton, CA, USA). Plasma glucose levels were measured using the glucose oxidase–peroxidase method (Wako Diagnostics, Tokyo, Japan). HbA1c levels were determined using boronate-affinity high-performance liquid chromatography (NGSP-certified, Primus Corp., Kansas City, MO, USA). AhR levels were determined using the quantitative sandwich enzyme immunoassay technique (Cusabio, Wuhan, China). The coefficient of variability of intra-assay precision for AhR was <8% on the basis of twenty tests on one plate, and the intra-assay CV was <10%. The eGFR was calculated according to the Chronic Kidney Disease Epidemiology Collaboration equation for Chinese individuals with type 2 diabetes [43]. The reference values of clinical variables are defined as follows: obesity was defined as a body mass index (BMI) ≥ 27 kg/m^2^ [44]; CKD was defined as an eGFR < 60 mL/min per 1.73 m^2^; an increased UACR was defined as a condition with a UACR ≥ 30 mg/g [45]; hypertension was defined as a systolic BP ≥130 mmHg, a diastolic BP ≥ 80 mmHg, or the documented use of antihypertensive medication; CAD was defined as a documented history of myocardial infarction or coronary revascularization; poor fasting glucose control was defined as a fasting glucose level ≥ 7.2 mmol/L (130 mg/dL); hypercholesterolemia was defined as a total cholesterol level ≥ 4.1 mmol/L (160 mg/dL) or an LDL level ≥ 2.6 mmol/L (100 mg/dL); and hypertriglyceridemia was defined as a triglyceride level ≥ 1.7 mmol/L (150 mg/dL) [46].

### 4.3. Statistical Analysis

All the continuous data are presented as the medians (interquartile ranges), and categorical data are presented as the numbers (percentages). A Mann–Whitney U test was performed to examine the intergroup differences in continuous variables. The differences in categorical variables were examined using either the chi-square test or Fisher’s exact test, as appropriate. ROC curve analysis was performed to differentiate a QFT-positive result based on serum AhR levels. Logistic regression analyses were used to estimate the OR and 95% CI of the factors associated with the positive QFT result; the potential confounding variables defined as the significant risk factor associated with both a positive QFT result and serum AhR levels were included in the multivariable model. The statistical power could reach 0.85 in the present study with an effect size of 0.3 for serum AhR levels between patients with and without positive QFT results. Statistical analysis was performed using SPSS version 22.0 software (IBM Corp., Armonk, NY, USA).

## 5. Conclusions

LTBI, confirmed by a QFT-positive result, is associated with an increase in serum AhR levels in patients with type 2 diabetes. Further large-scale studies and longitudinal follow-up for treatment of LTBI are needed.

## Figures and Tables

**Table 1:** Characteristics of enrolled patients categorized by the results of the QFT.

	QFT-Positive (*n* = 133)	QFT-Negative (*n* = 410)	*p*
Age (year)	66.0 (60.0, 72.0)	61.0 (55.0, 68.0)	<0.001 *
Male, *n* (%)	81 (60.9%)	228 (55.6%)	0.332
Hypertension, *n* (%)	106 (79.7%)	308 (75.1%)	0.337
Current smoker, *n* (%)	18 (13.5%)	39 (9.5%)	0.249
CAD history, *n* (%)	16 (12.0%)	35 (8.5%)	0.303
Diabetes duration (years)	13.0 (8.0, 20.0)	12.0 (7.0, 17.0)	0.024 *
BMI (kg/m^2^)	25.7 (23.3, 28.1)	25.7 (23.1, 28.5)	0.902
Systolic BP (mmHg)	132.0 (123.5, 146.0)	130.0 (120.0, 141.0)	0.066
Diastolic BP (mmHg)	74.0 (65.0, 80.0)	74.0 (65.0, 80.0)	0.480
Fasting glucose (mmol/L)	8.1 (6.5, 9.9)	8.2 (6.7, 10.2)	0.361
HbA1c (%)	8.4 (7.6, 9.7)	8.4 (7.4, 9.4)	0.705
Total cholesterol (mmol/L)	3.9 (3.3, 4.5)	4.0 (3.5, 4.6)	0.096
LDL cholesterol (mmol/L)	2.1 (1.7, 2.6)	2.2 (1.8, 2.8)	0.049 *
HDL cholesterol (mmol/L)	1.2 (1.0, 1.4)	1.2 (1.0, 1.5)	0.352
Triglycerides (mmol/L)	1.3 (0.9, 2.1)	1.4 (0.9, 2.0)	0.638
ALT (U/L)	22.0 (14.5, 31.0)	21.0 (15.0, 32.0)	0.789
C-reactive protein (mg/L)	1.0 (0.5, 1.8)	1.1 (0.5, 2.5)	0.084
eGFR (mL/min/1.73 m^2^)	67.0 (53.6, 81.5)	71.9 (59.8, 85.8)	0.001 *
Increased UACR	68 (51.1%)	195 (47.6%)	0.538
Use of antiplatelet agent, *n* (%)	24 (18.0%)	54 (13.2%)	0.211
Use of statins, *n* (%)	78 (58.6%)	251 (61.2%)	0.670
Use of antihypertensive medications, *n* (%)	55 (41.4%)	159 (38.8%)	0.670
ACE inhibitors or ARBs, *n* (%)	41 (30.8%)	143 (34.9%)	0.452
α-blockers, *n* (%)	4 (3.0%)	12 (2.9%)	0.999 ^F^
β-blockers, *n* (%)	9 (6.8%)	25 (6.1%)	0.943
Calcium channel blockers, *n* (%)	25 (18.8%)	59 (14.4%)	0.279
Diuretics, *n* (%)	12 (9.0%)	29 (7.1%)	0.408
Use of insulin therapy, *n* (%)	67 (50.4%)	195 (47.6%)	0.642
Use of GLP-1 RAs, *n* (%)	7 (5.3%)	32 (7.8%)	0.428
Use of oral glucose-lowering medications, *n* (%)	119 (89.5%)	368 (89.8%)	0.999
Sulfonylurea, *n* (%)	43 (32.3%)	178 (43.4%)	0.031 *
Glinides, *n* (%)	17 (12.8%)	29 (7.1%)	0.061
Metformin, *n* (%)	86 (64.7%)	278 (67.8%)	0.649
DPP4 inhibitors, *n* (%)	76 (57.1%)	202 (49.3%)	0.139
SGLT2 inhibitors, *n* (%)	12 (9.0%)	68 (16.6%)	0.046 *
Thiazolidinediones, *n* (%)	27 (20.3%)	100 (24.4%)	0.395
α-glucosidase inhibitors, *n* (%)	10 (7.5%)	16 (3.9%)	0.143

Continuous data are presented as the medians (interquartile ranges), and categorical data are presented as the numbers (percentages). *: *p*-value < 0.05. ^F^: Fisher’s exact test due to the expected value of <5 for the patients using α-blockers in the QFT-positive group. Abbreviations: ACE = angiotensin-converting enzyme, ALT = alanine aminotransferase, ARB = angiotensin II receptor antagonist, BMI = body mass index, BP = blood pressure, CAD = coronary artery disease, DPP4 = dipeptidyl peptidase-4, eGFR = estimated glomerular filtration rate, GLP-1 RAs = glucagon-like peptide-1 receptor agonists, HbA1c = glycated hemoglobin, HDL = high-density lipoprotein, LDL = low-density lipoprotein, QFT = QuantiFERON-TB, SGLT2 = sodium glucose cotransporter 2, UACR = urine albumin-to-creatinine ratio.

**Table 2:** Medians (interquartile ranges) of serum AhR levels in the enrolled patients categorized by associated factors.

	Group	Case Number	Median	IQR (25%, 75%)	*p*
Age	<63years ^†^	271	37.5	(17.4, 54.3)	0.013 *
	≥63years ^†^	272	42.6	(21.5, 58.1)	
Sex	Female	234	41.1	(18.2, 58.1)	0.856
	Male	309	39.6	(21.3, 55.7)	
Hypertension	No	129	39.3	(14.3, 51.5)	0.117
	Yes	414	41.0	(20.7, 57.3)	
Current smoker	No	486	40.2	(18.7, 56.8)	0.769
	Yes	57	41.6	(28.6, 52.9)	
CAD history	No	492	40.4	(19.1, 57.2)	0.446
	Yes	51	40.9	(25.7, 48.2)	
Diabetes duration	<13 years ^†^	265	39.3	(20.7, 55.9)	0.737
	≥13 years ^†^	278	41.7	(19.2, 56.5)	
BMI	<27 kg/m^2^	339	41.0	(21.2, 56.2)	0.634
	≥27 kg/m^2^	204	39.2	(17.6, 56.6)	
Systolic BP	<130 mmHg	234	38.0	(18.8, 55.4)	0.134
	≥130 mmHg	309	41.9	(20.4, 56.9)	
Diastolic BP	<80 mmHg	369	40.7	(19.5, 56.1)	0.741
	≥80 mmHg	174	39.5	(19.9, 56.7)	
Fasting glucose	<7.2 mmol/L	187	38.6	(18.6, 57.6)	0.561
	≥7.2 mmol/L	356	41.2	(20.5, 55.4)	
HbA1c	<8.4% ^†^	265	39.5	(18.3, 54.4)	0.178
	≥8.4% ^†^	278	41.4	(20.8, 57.9)	
Total cholesterol	<4.1 mmol/L	303	41.8	(20.5, 56.2)	0.390
	≥4.1 mmol/L	240	39.2	(17.7, 56.5)	
LDL cholesterol	<2.6 mmol/L	377	40.3	(19.9, 56.5)	0.835
	≥2.6 mmol/L	166	40.8	(18.5, 55.4)	
Low HDL cholesterol	No	349	40.9	(16.9, 57.1)	0.823
	Yes	194	39.5	(21.8, 55.2)	
Triglycerides	<1.7 mmol/L	343	41.0	(17.4, 55.8)	0.632
	≥1.7 mmol/L	200	38.6	(22.7, 56.7)	
ALT	<21 U/L ^†^	224	40.6	(19.2, 55.7)	0.659
	≥21 U/L ^†^	319	40.2	(20.3, 56.9)	
C-reactive protein	<1.11 mg/L ^†^	272	39.5	(20.5, 55.8)	0.935
	≥1.11 mg/L ^†^	271	41.6	(18.6, 56.5)	
eGFR (mL/min/1.73 m^2^)	≥60	392	39.4	(18.0, 55.1)	0.043 *
	<60	151	42.8	(22.9, 59.2)	
UACR	<30 mg/g	280	39.7	(16.8, 54.3)	0.032 *
	≥30 mg/g	263	42.1	(21.9, 58.7)	
Use of antiplatelet	No	465	40.7	(20.4, 56.6)	0.555
	Yes	78	39.2	(16.0, 55.0)	
Use of statins	No	214	44.3	(23.6, 58.1)	0.013 *
	Yes	329	38.4	(17.4, 55.0)	
Use of antihypertensive drugs	No	329	40.5	(18.2, 55.5)	0.360
	Yes	214	40.4	(20.8, 57.7)	
ACE inhibitors or ARBs	No	359	40.5	(18.8, 55.5)	0.293
	Yes	184	40.4	(20.8, 58.4)	
α-blockers	No	527	40.6	(20.3, 56.2)	0.808
	Yes	16	38.5	(12.4, 58.4)	
β-blockers	No	509	40.3	(19.9, 56.0)	0.496
	Yes	34	46.3	(17.9, 57.6)	
Calcium channel blockers	No	459	40.6	(20.3, 55.9)	0.970
	Yes	84	39.4	(18.7, 56.7)	
Diuretics	No	501	40.2	(19.4, 55.7)	0.259
	Yes	42	43.2	(23.3, 58.9)	
Use of insulin therapy	No	281	39.9	(18.9, 55.0)	0.542
	Yes	262	41.0	(20.4, 58.3)	
Use of GLP-1 RAs	No	504	40.8	(20.8, 56.6)	0.251
	Yes	39	35.8	(12.3, 55.4)	
Use of oral glucose-lowering medications	No	56	33.6	(13.7, 53.2)	0.209
	Yes	487	40.9	(20.3, 56.6)	
Sulfonylurea	No	322	38.7	(18.8, 55.4)	0.130
	Yes	221	42.5	(20.3, 58.4)	
Glinides	No	497	40.2	(19.5, 55.8)	0.242
	Yes	46	45.2	(20.6, 60.4)	
Metformin	No	173	44.4	(21.2, 59.2)	0.126
	Yes	370	39.3	(18.7, 55.4)	
DPP4 inhibitors	No	265	37.5	(17.4, 54.3)	0.017 *
	Yes	278	42.3	(23.6, 58.3)	
SGLT2 inhibitors	No	463	41.0	(20.8, 57.0)	0.130
	Yes	80	38.0	(14.5, 50.6)	
Thiazolidinediones	No	416	41.2	(19.3, 57.6)	0.580
	Yes	127	37.6	(20.8, 54.4)	
α-Glucosidase inhibitors	No	517	40.5	(19.9, 56.3)	0.994
	Yes	26	36.7	(17.9, 58.6)	

^†^: Categorized by median of variables. *: *p*-value < 0.05. Abbreviations: ACE = angiotensin-converting enzyme, AhR = aryl hydrocarbon receptor, ALT = alanine aminotransferase, ARB = angiotensin II receptor antagonist, BMI = body mass index, BP = blood pressure, CAD = coronary artery disease, DPP4 = dipeptidyl peptidase-4, eGFR = estimated glomerular filtration rate, GLP-1 RAs = glucagon-like peptide-1 receptor agonists, HbA1c = glycated hemoglobin, HDL = high-density lipoprotein, LDL = low-density lipoprotein, SGLT2 = sodium glucose cotransporter 2, UACR = urine albumin-to-creatinine ratio.

**Table 3:** Multivariable logistic regression analysis showing the factors associated with positive QFT results.

	Crude Model			Multivariable Model		
	OR	95% CI	*p*	OR	95% CI	*p*
AhR ≥ 37.7 pg/mL	2.023	(1.343, 3.047)	<0.001	1.902	(1.254, 2.886)	0.003
Age ≥ 63 years				2.258	(1.454, 3.507)	<0.001
CKD				1.209	(0.768, 1.904)	0.412

Abbreviations: AhR = aryl hydrocarbon receptor, CI: confidence interval, CKD = chronic kidney disease, OR = odds ratio, QFT = QuantiFERON-TB.

**Table 4:** Serum AhR level was an independent risk factor for QFT-positive results in patients stratified by age (63 years).

	Age < 63 Years (*n* = 271)			Age ≥ 63 Years (*n* = 272)		
	OR	95% CI	*p*	OR	95% CI	*p*
AhR ≥ 37.7 pg/mL	2.218	(1.126, 4.371)	0.021	1.717	(1.010, 2.918)	0.046
CKD	1.910	(0.783, 4.655)	0.155	1.048	(0.627, 1.752)	0.857

Abbreviations: AhR = aryl hydrocarbon receptor, CKD = chronic kidney disease, QFT = QuantiFERON-TB.

## Data Availability

The data presented in this study are available on request from the corresponding author.

## Abbreviations

The following abbreviations are used in this manuscript:

AHAS	acetohydroxyacid synthase
AhR	aryl hydrocarbon receptor
ALT	alanine aminotransferase
ARNT	ahR nuclear translocator
BMI	body mass index
BP	blood pressure
CAD	coronary artery disease
CI	confidence interval
CKD	chronic kidney disease
CRP	C-reactive protein
CVD	cardiovascular disease
DPP4	dipeptidyl peptidase-4
eGFR	estimated glomerular filtration rate
GLP-1 RAs	glucagon-like peptide-1 receptor agonists
HbA1c	glycated hemoglobin
HDL	high-density lipoprotein
IL	interleukin
IR	Insulin resistance
LTBI	latent tuberculosis infection
Mtb	mycobacterium tuberculosis
OR	odds ratio
QFT	QuantiFERON-TB
QFT-GIT	QuantiFERON-TB Gold In-Tube
ROC	receiver operating characteristic
SGLT2	sodium–glucose cotransporter 2
TB	tuberculosis
TNF	tumor necrosis factor
UACR	urine albumin–creatinine ratio
